# Autoimmune Encephalitis in the Philippines: A Scoping Review on the Treatment Gaps, Challenges, and Current State of Care

**DOI:** 10.3389/fneur.2022.788309

**Published:** 2022-02-07

**Authors:** Gerald T. Pagaling, Christian Wilson R. Turalde, Roland Dominic G. Jamora

**Affiliations:** ^1^Department of Neurosciences, College of Medicine and Philippine General Hospital, University of the Philippines Manila, Manila, Philippines; ^2^Institute for Neurosciences, St. Luke's Medical Center, Quezon City, Philippines

**Keywords:** autoimmune encephalitis, Philippines, management, treatment, challenges

## Abstract

**Objectives:**

We reviewed the current health service delivery for individuals with Autoimmune Encephalitis (AE) in the Philippines and to identify the gaps and challenges in its management.

**Methodology:**

We conducted a scoping review of pertinent literature AE in the Philippines using the Preferred Reporting Items for Systematic reviews and Meta-analysis (PRISMA) guidelines. We extracted data on epidemiology, legislation, health financing, information systems, pharmacotherapy, and healthcare services related to the management of AE in the local setting.

**Discussion:**

The epidemiology of AE is still unknown. Out-of-pocket expenses contribute to most of the healthcare expenditure despite government-led programs to reduce the financial burden. The access to diagnostic examinations such as magnetic resonance imaging, electroencephalogram, and antibody testing is limited by the geographic distribution of the facilities and costs. The acute and long-term management of AE are cost-prohibitive and are not readily available. There are significant treatment gaps in the care of individuals with AE in the Philippines in terms of disease recognition, resource allocation, access to satisfactory diagnostic evaluation, and provision of prognosis-changing therapeutics. We proposed core strategies that can address these treatment gaps such as increasing awareness, improving access to health resources, adequate healthcare financing, and availability of support systems.

## Introduction

Autoimmune encephalitis (AE) is a non-infectious or para-infectious immune-mediated inflammatory disorder of the brain parenchyma, leptomeninges, and cerebral vessels commonly presenting with subacute onset alteration in mental status, memory deficits, or psychiatric symptoms accompanied by a variety of neurologic findings (1–4). Neurologic findings can be a focal central nervous system lesion, seizures, cerebrospinal fluid (CSF) pleocytosis, magnetic resonance imaging (MRI) features such as hyperintense T2-weighted fluid-attenuated inversion recovery signals in medial temporal lobes and multifocal inflammatory demyelinating areas of the gray and/or white matter (1, 2).

Recent epidemiologic studies suggest that AE has an estimated prevalence rate of 13.7/100,000 (3). The recent advances in the identification of antibodies and their associated syndromes can further uncover the real prevalence rate of AE as under-reporting of this disorder is relatively common (2). However, the rapidly expanding knowledge on AE in both laboratory and clinical practice led to the heterogeneity of data regarding its clinical picture creating gaps in knowledge and practice. In particular, the diagnosis of autoimmune encephalitis necessitates diagnostic tests such as cranial magnetic resonance imaging (MRI), electroencephalogram (EEG), and cerebrospinal (CSF) studies that are not easily accessible (1, 2). Furthermore, the best available treatments of AE such as intravenous immunoglobulin (IVIG) administration, methylprednisolone pulse therapy (MPPT), plasma exchange (PLEX), and other immunosuppressive agents are also cost-prohibitive and are not readily available (5). The advances in the diagnosis and treatment of AE are usually available in high-income countries. The overall burden of AE is yet to be determined, however in general, the burden of neurologic diseases is high especially in low- to middle-income countries due to lack of funding allocated for health (6). The Philippines is categorized under the lower-middle-income countries with a population of 110 million and is labeled as one of the countries with the highest disability-adjusted life years per 100,000 population (7, 8). The burden of AE however is not yet established in the Philippines. To date, no comprehensive report has been made on the treatment gaps and challenges in AE care in the Philippines. We aimed to identify and evaluate the treatment gaps in the management of AE in the Philippines through systematic literature search and review of relevant Philippine websites.

## Materials and Methods

### Protocol

Our study adhered to the Preferred Reporting Items for Systematic reviews and Meta-analysis (PRISMA) guidelines extension for the scoping review (9).

### Eligibility Criteria for Including Studies in the Scoping Review

We considered published guidelines, meta-analyses, systematic reviews, review articles, randomized controlled trials, prospective/retrospective cohort studies, case series and reports, abstracts, conference proceedings, editorials, and textbooks with authors affiliated with an institution in the Philippines or with the study set in the Philippines. Human (both pediatric and adult population) and animal studies were included. Articles not in English or Filipino were excluded. No restriction in terms of the date of publication was implemented.

### Information Sources

We searched international (PubMed, Scopus, Clinicaltrials.gov, Ebscohost, Western Pacific Region Index Medicus, and Web of Science) and local (Health Research and Development Information Network) medical databases for relevant studies. We accessed pertinent and available literature via official websites and/or email correspondence with the following: international organizations, government and non-government agencies [Philippine Health Insurance Corporation (Philhealth), Department of Health (DOH), Philippine Statistics Authority], medical associations (Philippine Neurological Association, Autoimmune Encephalitis Clinicians Network, Philippine Academy of Rehabilitation Medicine**)**, and private organizations (clinical laboratories and pharmaceutical companies).

### Search Selection of Sources

We conducted a scoping review of literature from the earliest indexed record of the databases up to October 2021 using the following search term strategy that includes paraneoplastic and nonparaneoplastic cases: Autoimmune encephalitis OR autoimmune epilepsy OR Anti-Hu encephalitis OR Anti-Ma2 encephalitis OR AntiAMPAR encephalitis OR AntiLGI1 encephalitis OR AntiCASPR2 encephalitis OR AntiGAD65 encephalitis OR AntiGABAxA encephalitis OR AntiGABAxB encephalitis OR AntiDPPX encephalitis OR AntimGluR5 encephalitis OR AntiAK5 encephalitis OR AntixNeurexine-3a encephalitis OR AntiPCA2 encephalitis OR AntiMAP1b encephalitis OR AntiNMDAR encephalitis OR AntiMOG encephalitis OR AntiCRMP5 encephalitis OR AntiCV2 encephalitis OR AntiRi encephalitis OR AntiKHL11 encephalitis OR AntiIgLON5 encephalitis OR AntiVGKC encephalitis OR AntiNIF encephalitis OR AntiGFAP encephalitis OR Glycine receptor encephalitis OR AntiAmphyphysin encephalitis OR seronegative autoimmune encephalitis AND Philippines. All titles and available abstracts were screened based on the eligibility criteria. GTP and CWT separately searched available literature. Duplicates were excluded. We retrieved the full-text eligible articles for data extraction.

### Data Charting Process and Items

We extracted data on epidemiology, legislation, health financing, information systems, pharmacotherapy, and healthcare services related to the management of AE in the local setting. Details on authors, titles, and institutional affiliation were extracted from published studies. For the information on the specific cost of diagnostics and medications, communications were done with relevant laboratories and institutions.

## Results

### The Search of Studies

**Figure 1:**
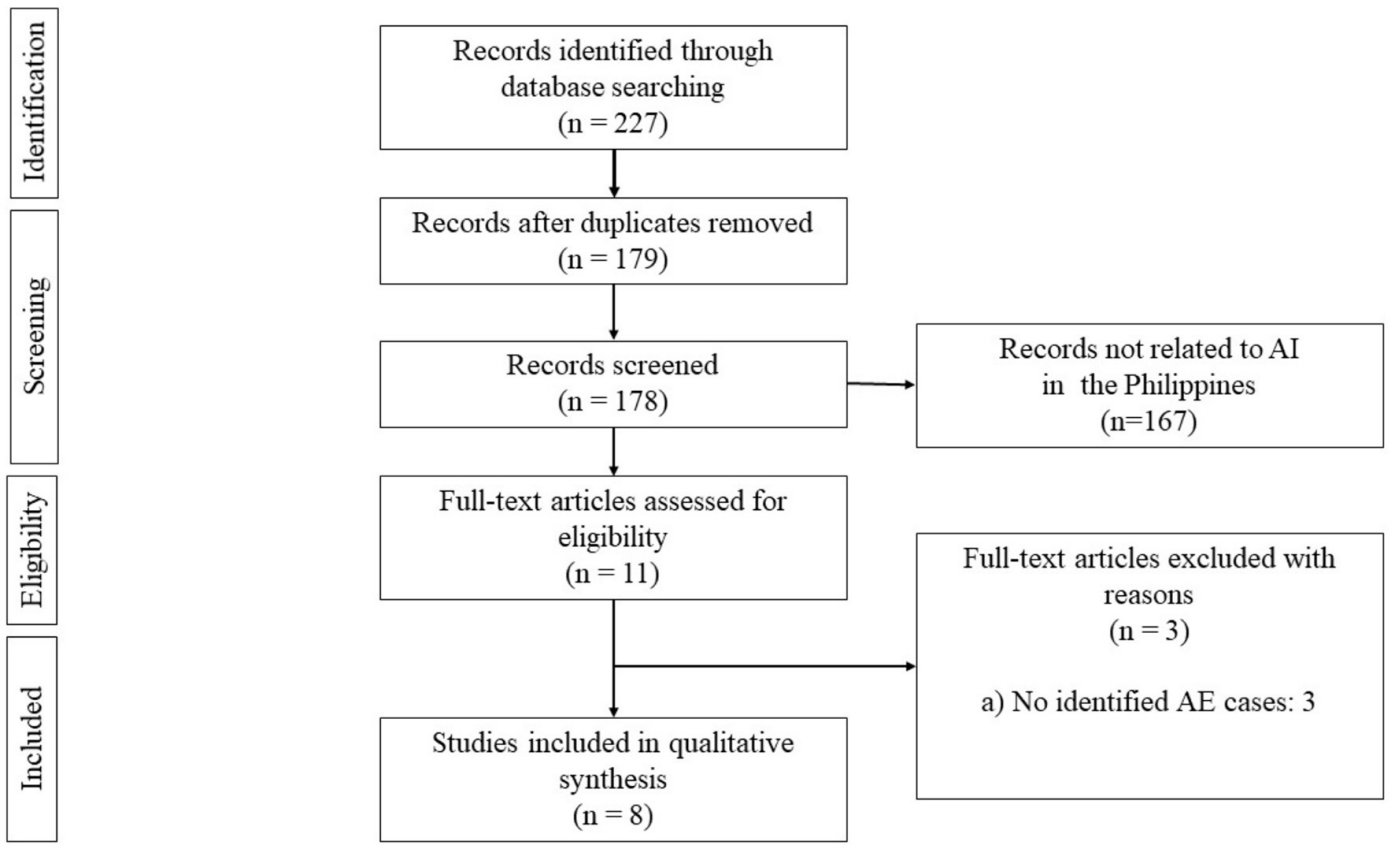
Flow diagram adapted from PRISMA guidelines for scoping reviews.

The search yielded a total of 181 articles (Figure 1). We then screened 171 articles after the duplicates were removed. We excluded 161 articles that were not related to AE in the Philippines. Ten articles were assessed for eligibility, and we excluded four articles with no identified AE cases. Thus, a total of six data sources were included for synthesis.

### The National Acute Meningitis-Encephalitis Syndrome Surveillance and Case Registry

In 2014, the Epidemiology Bureau mandated the establishment of a national surveillance system for acute encephalitis syndrome and meningitis. This encompasses all cases of bacterial meningitis, Japanese encephalitis, and encephalitides with pending work-up across all age groups in the country within nine sentinel hospitals (10). Last 2020 there were 1,437 identified cases with 77 recorded deaths (11). Last 2017, there were 778 suspected cases, 471 cases underwent testing and 57 cases tested positive for a specific etiologic agent. The identified cases were Japanese encephalitis virus, Streptococcus pneumoniae, Micrococcus, Neisseria meningitides, Haemophilus influenza, Dengue, Staphylococcus epidermidis, Streptococcus agalactiae, Pseudomonas, and Escherichia coli (12). It can be observed that only 60.5% of the labeled cases of meningitis-encephalitis in the Philippines underwent laboratory testing and only 7.33% cases have an identified etiology (12). The latest report did not further elaborate if the other identified cases have undergone additional testing such as work-up for autoimmune encephalitis (12). Although there were no publicly available individual data on the reported cases, the diagnoses of 92% of the cases remain to be identified. In the updated International Case Definition−10 (ICD-10) medical case rates in the Philippines, autoimmune encephalitis is not yet recognized as a diagnosis and only falls under the category “Encephalitis, myelitis, and encephalomyelitis, unspecified or Encephalitis, myelitis, and encephalomyelitis in other disease classified elsewhere” which limits proper reporting of identified cases (13).

Most of the hospitals in the Philippines still rely on printed medical records and only a small proportion of tertiary hospitals using electronic medical records. The law mandates that the electronic and non-electronic medical records are stored for only fifteen years (14). However, the retrieval of physical medical records for review can also prove to be cumbersome as proper record keeping is limited by the resources available in each institution such as quality of the papers, storage capacity of records division, and efficiency if archiving systems.

### Epidemiology of AE in the Philippines

In a retrospective study done in 2020 that reviewed 18 cases (12 adults and six children) of patients with AE admitted to the Philippine General Hospital (PGH), the largest tertiary hospital providing neurologic care in the Philippines, the median age of onset was 13 years among pediatric cases and 32 years in the adult with a male-female ratio of 1:2.6 (15). Anti-NMDAR antibody AE was the most common type in adults (58.3%) and children (100%) and other documented syndromes had antibodies against alpha-amino-3-hydroxy-5-methyl-4-isoxazole propionic acid receptor (AMPAR), voltage-gated potassium channel complex (VGKC-complex), and multiple antibodies (NMDAR + AMPAR, NMDAR + VGKC) (15). The diagnosed cases with anti-VGKC antibodies still used this nomenclature since the identification of specific antibodies to the anti-VGKC namely leucine-rich glioma-inactivated (LGI1) and contactin-associated protein-like 2 (CASPR2) were not yet implemented. EEG was done in 17 of the patients where 16 had abnormalities with five adults presenting with status epilepticus (SE) (15). In terms of antiseizure treatment, 14 needed polytherapy and only three were on monotherapy (15). In terms of immunotherapy, 17 received first-line immunotherapy, nine had MMPT and IVIG, six had MPPT only, one had high dose MPPT and PLEX, and one had IVIG alone. For the second line immunotherapy, two had cyclophosphamide, one had rituximab, and one had rituximab and cyclophosphamide (15). There is still a deficiency in the updated and local epidemiological data on AE in the Philippines as there is still no existing national registry program for AE (16). Table 1 summarizes the clinical profile and outcomes of the identified AE cases in the Philippines from published data.

**Table 1:** Summary of clinical profile and outcomes of identified autoimmune encephalitis cases in the Philippines from published data.

**Case**	**Age/sex**	**Implicated antibody/ies**	**Clinical presentation and associated neoplasm**	**Electro-encephalo-graphic findings**	**Treatment**	**Outcome**	**Reference**
1	25/F	NMDAR Ab, VGKC Ab	Behavioral changes, seizures, orofacial and limb dyskinesias, associated with ovarian teratoma	Electrographic seizures arising from the right fronto-temporal region	Surgery, MPPT, IVIG	Improved	Reyes et al. (17)
2	43/F	NMDAR Ab	Behavioral changes, mutism, seizures, neuroleptic malignant syndrome	*Not mentioned*	*Not mentioned*	*Not mentioned*	Señga et al. (18)
3	46/F	VGKC Ab	Behavioral changes, seizures, memory loss	Intermittent slowing on bilateral frontotemporal regions, no epileptiform discharges	MPPT	Improved; able to perform basic activities of daily living	Ocampo et al. (19)
4	36/F	NMDAR Ab, AMPAR Ab	Behavioral changes, hallucinations, orofacial dyskinesia, hyperekplexia, ovarian teratoma	Slow activity	Surgery, MPPT, IVIG, RTX	Did not improve	Reyes et al. (15)
5	24/F	NMDAR Ab	Generalized seizures, hallucinations, orofacial dyskinesias	Slow activity	MPPT, IVIG	Improved	
6	51/M	VGKC Ab	Focal seizures, behavioral changes, memory lapses, autonomic instability	Slow activity, epileptic activity	MPPT	Did not improve	
7	32/F	NMDAR Ab	Memory lapses, hallucinations, mania, anhedonia, seizure, orofacial and limb dyskinesias, ovarian teratoma	Slow activity, epileptic activity	Surgery, MPPT	Improved	
8	56/M	NMDAR Ab	Speech disturbances, echolalia, perseveration, anxiety, agitation, hallucinations, aggression, memory lapses	Slow activity	MPPT, IVIG	Improved	
9	44/F	AMPAR Ab	Memory lapses, hallucinations, aggression, catatonia, mutism, status epilepticus, orofacial and limb dyskinesias	Not done	MPPT	Did not improve	
10	25/M	NMDAR Ab	Speech disturbances, generalized seizure, orofacial dyskinesias, autonomic instability	Slow activity	MPPT, IVIG, RTX, CYC	Mortality	
11	29/M	NMDAR Ab	Hallucinations, catatonia, anxiety, speech disturbances, tremors	Slow activity	MPPT	Improved	
12	32/F	VGKC Ab	Seizure, status epilepticus, autonomic instability	Slow activity, epileptic activity	MPPT, IVIG	Did not improve	
13	28/F	NMDAR Ab	Seizures, orofacial dyskinesias, autonomic instability, ovarian teratoma	Slow activity	Surgery, MPPT, IVIG	Improved	
14	33/F	NMDAR Ab	Hallucinations, paranoia, depressed mood, tremors, autonomic instability	Slow activity	No treatment	Mortality	
15	10/F	NMDAR Ab	Talkativeness, incoherent responses, depressed mood, seizures, orofacial dyskinesias,	Excessive beta activity	MPPT, IVIG, CYC	Improved	
16	18/F	NMDAR Ab	Seizures, orofacial dyskinesias	Slow activity	MPPT	Improved	
17	7/F	NMDAR Ab	Hallucinations, memory lapses, status epilepticus, autonomic instability, ovarian teratoma	Slow activity	Surgery, IVIG	Improved	
18	11/F	NMDAR Ab	Seizures, orofacial and limb dyskinesias, limb dystonia, athetosis, autonomic instability	Normal findings	MPPT, IVIG	Improved	
19	15/F	NMDAR Ab	Paranoia, aggression, focal seizures, insomnia, orofacial dyskinesias	Slow activity	MPPT, IVIG	Improved	
20	15/M	NMDAR Ab	Hallucinations, orofacial dyskinesias	Slow activity	MPPT, CYC	Improved	

*Ab, antibody; AMPAR, α-Amino-3-hydroxy-5-methyl-4-isoxazolepropionic acid receptor; CYC, cyclophosphamide; F, female; IVIG, intravenous immunoglobulin; M, male; MPPT, methylprednisolone pulse therapy; NMDAR, N-methyl-D-aspartate receptor; RTX, rituximab; VGKC - voltage-gated potassium channel*.

### Local Research in AE

As of November 2021, there were only six indexed research outputs from the Philippines (Table 2): three case reports, two retrospective case series, and one abstract of a case report. Four studies were published in the last decade by authors affiliated with PGH.

**Table 2:** Indexed research output on autoimmune encephalitis from the Philippines.

**Title**	**Author**	**Affiliation**	**Journal**	**Description**
Autoimmune encephalitis in a tertiary hospital in the Philippines	Reyes et al. (15)	Philippine General Hospital Manila, Philippines	Journal of clinical neuroscience	Retrospective cohort
Autoimmune encephalitis associated with two antibodies	Reyes et al. (17)	Philippine General Hospital Manila, Philippines	Epilepsy and behavior	Case report
Neuroleptic malignant syndrome in anti-NMDAR encephalitis–a phenomenon of delayed recognition and psychotropic drug resistance	Senga and Reyes (18)	The Medical City Pasig City, Philippines	Journal of the neurological sciences	Case report-abstract
Clinical features and outcomes of nonconvulsive status epilepticus in a developing country: a 5-year retrospective study	Andal et al. (20)	Philippine General Hospital Manila, Philippines	Epilepsy and behavior	Retrospective cohort
Electroencephalographic findings in antileucine-rich glioma-inactivated 1 (LGI1) autoimmune encephalitis: a systematic review	Roberto et al. (21)	Philippine General Hospital Manila, Philippines	Epilepsy and behavior	Systematic review
Voltage-gated potassium channel complex (VGKC) antibody encephalitis in a Filipino adult: a reversible cause of early-onset neurocognitive disorder	Ocampo et al. (19)	St. Luke's Medical Center Quezon City, Philippines	Clinical and experimental neuroimmunology	Case report

### The Philippine Healthcare System

The Philippines is an archipelago of 7,107 islands stretched across a land area of 298,170 square kilometers with a population of around 109 million as of 2020 (7, 22). The healthcare delivery system in the Philippines is divided into public and private sectors. The DOH handles national health policies and services while the local government units (LGU) are in charge of local service delivery and policy implementations (22). This devolution is dependent on the assumption that the LGUs are more abreast with the situation of their constituents and can promptly adapt to their dynamic needs (22). However, due to the topography of the Philippines, some rural areas are inevitably isolated rendering them disadvantaged in availing healthcare (22). Furthermore, this lack of access prevents the collection of population-specific data that will address the needs of the community (22, 23).

### Healthcare Cost and Coverage in the Philippines

In 2019, the country's health care expenditure reached USD 15.9 billion (USD 1 = PHP 49.68 as of September 1, 2021), 47.9% of this came from out-of-pocket payment while the government health care financing shoulders around 42.0% and the rest were covered from voluntary health care payments (24). However, in the past 20 years, the government's contribution to the health care expenditure has been decreasing from 37.34% in 2000 to only 29.29% in 2018 (25). Conversely, the proportion of out-of-pocket expenditure in health has increased from 41.19% in 2000 to 52.85% in 2018 (25). The government healthcare financing is largely dependent on a tax-based budgeting system that funds government-led facilities throughout the country (22). Other government-led programs were developed to expand the coverage of health expenditure such as the PhilHealth and the *Malasakit Center* (26, 27). However, these only cover expenses upon hospital admission, and outpatient expenses from consultations, diagnostic examinations, and medications are usually paid on an out-of-pocket basis (26, 27). Moreover, only 66% of the Filipino household population has any form of Philhealth and only 24% have other forms of public and private health insurances (28). Other measures to alleviate financial burden include the Magna Carta for Disabled Persons that entitles persons with disabilities to certain benefits including equal opportunity for employment, access to quality education, discounts for healthcare-related expenses (29). Autommine encephalitis is currently not listed in the list of medical cases covered by PhilHealth and can only fall under the category of other encephalitis not otherwise specified with a case rate of USD 432 (13). There are also non-government-led funding organizations that cater to patients with neurologic disease such as the Let's Save the Brain Foundation that can be tapped to provide support (diagnostic evaluation and treatment regimens) for patients with AE admitted at PGH (30).

### Specialty Training and Continuing Medical Education

As of this writing, there are 508 active neurologist in the country, 421 adult neurologists, 83 pediatric neurologist, four adult and pediatric neurologists, one neurologist specializing in neuroimmunology, and two are members (pediatric neurologists) of the Autoimmune Encephalitis Clinicians Network (31). Approximately, one adult neurologist caters to 176,000 adult Filipinos or 0.57 adult neurologist to 100,000 adult population and one pediatric neurologist to 299,581 Filipino children or 0.33 pediatric neurologist to 100,000 pediatric population. This is below the World Health Organization recommendation of 1–5 neurologists per 100,000 population (6). The adult neurology training in the country takes 3–4 years including training in internal medicine. The pediatric neurology fellowship takes 3 years. As of writing, there are only 11 accredited training institutions offering adult neurology residency; nine of which are in Manila, the capital (31). Subspecialty training in neuroimmunology is currently not available in the country. Due to the relatively new practice of neuroimmunology, there are still no programs aimed toward awareness and education of general practitioners and neurologists regarding autoimmune encephalitis.

### Challenges in Diagnosis

The diagnosis of AE requires early extensive workup such as cranial MRI, EEG, CSF studies, fluorodeoxyglucose positron emission tomography (FDG-PET), and serum analysis (1, 2, 32).

As of 2016, there are only around 78 MRI units (0.8 MRI units per online million population) in the country operated by public and private sectors, and the majority are located in Manila (22). Cranial MRI with contrast usually costs around USD 103–618 (33). There are only seven institutions that offer FDG-PET scans in the country and the price ranges from USD 604–1000. There is still no data available on the number of EEG units in the country and the cost is around USD 60–150.

The recommended CSF analyses to rule out other differential diagnoses include cell counts, protein, glucose, immunoglobulin-G index, oligoclonal bands, viral panel, and culture studies (1, 2). CSF and serum antibody analysis usually confirm the inflammatory nature of encephalitis and the clinical recommendation is to send for the most comprehensive panel due to the significant overlap of clinical findings and antibody-negative cases (1, 2). The cost of the basic CSF analyses in the country ranges from USD 5–10. The only available AE antibody tests in the country are for antibodies against NMDA, Hu, Ma, and Ri which costs between USD 100–200 each, and the turnaround time is 3–4 weeks. CSF specimens are usually sent to diagnostic centers outside the country with a longer turnaround time. The comprehensive antibody tests are not available and are not covered in the benefit packages of Philhealth (13). Aside from the neurologic workup, it is also recommended to perform procedures to screen for cancer such as chest, abdominal, and pelvic CT scans, mammogram, and ultrasound since it is nearly impossible to predict if the encephalitis is paraneoplastic or non-paraneoplastic (2, 32).

In the local setting, immunologic procedures such as antibody tests are performed by licensed medical technologists under the supervision of board-certified pathologists (34). As of 2011, there were only 5,063 registered medical technologists in the country (medical technologist-to-population ratio of 1:18,238) (35). Despite the favorable healthcare professional-to-population ratio and the steady increase in the number of higher education institutions offering medical technology and medical laboratory science programs in the country (36), there is significant underutilization of medical technologists as majority of the laboratory facilities offer only minimal services such as acid-fast bacilli smears, urinalysis, and gram staining tests (37).

### Challenges in the Acute Treatment

The 2016 AE clinical diagnostic criteria stress the need to start immunotherapy if AE is highly suspected and infectious causes are ruled out (1). Currently, there are only several retrospective studies that showed early and aggressive immunotherapy has led to better outcomes (2, 32, 38). Anecdotal evidence includes the use of MPPT, IVIG, PLEX, and/or combination among the three as the first-line agents (2, 32, 38). Second-line agents such as rituximab and cyclophosphamide are recommended if there are no clinical or radiological responses to first-line therapy after 2–4 weeks (2, 32, 38). However, these treatment modalities are not readily available throughout the country, are cost-prohibitive, and are not included in the benefits package of Philhealth; hence financing is usually made as an out-of-pocket expense (13). The summary of the costs of treatment for AI in the Philippines is displayed in Table 3.

**Table 3:** Sample costing of treatment for autoimmune encephalitis in the Philippines (39).

**Drug name**	**Regimen**	Recommended price range based on 2020 DPRI (USD)^**^
**Available first-line immunosuppressive treatment**		
High dose pulse corticosteroids	IV methylprednisolone 1 gm OD x 5–7 days	206.00–517.00
Plasmapheresis (albumin)	Every other day for 5–10 sessions	1,509.66–6,622.40
IV immunoglobulin^*^	2 g/kg for 3–5 days	4,931.40–5,072.46
**Available second-line immunosuppressive treatment**		
Rituximab	1 gm IV once a week for 4 weeks	1,074.76
Cyclophosphamide^*^	600 mg/m^2^ IV every 4 weeks	2.51–3.98
**Other immunosuppressive regimens**		
Azathioprine 50 mg/tab	50–300 mg per day	562.10–1,693.60
Mycophenolate mofetil 100 mg/tab	500 mg/tab	324.00–477.55

*DPRI, Drug price reference index; IV, Intravenous; gm, gram; mg, milligram; OD, once a day*.

* *Sample computation based on a 60 kg and 160 cm person, with body surface area of 1.63/m^2^*.

** *USD 1.00 = PHP 49.68 as of September 1, 2021*.

### Challenges in Long-Term Immunosuppression

As per the recent treatment recommendations, acute therapy is followed by a gradual taper of immunosuppression (5, 32, 38). Some cases require long-term immunosuppression such as cases related to neuronal surface antibodies and different AE phenotypes (5). However, since there are no formal guidelines or randomized-controlled trials to identify which patients or type of AE will require long-term immunosuppression, it will be based on the clinical decision of the neurologist to pursue continued immunosuppression or not, what type of immunosuppressive regimen, and for how long (5, 38). Some of the most popular immunosuppressants used aside from rituximab are azathioprine and mycophenolate mofetil (5, 38). The optimal duration of immunosuppressive therapy in AE is unknown, however, arbitrary recommendations suggest three years followed by re-evaluation (5, 32).

### Support Group

There are currently no local support groups established in the Philippines for patients with AE. There are international networks for clinicians involved in the improvement of care for AE patients; however, only two Filipino neurologists are active members (40, 41).

### Rehabilitation Centers and Rehabilitation Medicine Specialists

The majority of patients with AE have residual signs and symptoms that require physical therapy, neurorehabilitation, and neuropsychological support (5). Furthermore, many patients will require evaluation of their functional capacity before resumption to their previous role. While their value has not been fully investigated, the outcomes of rehabilitation from clinical experiences are promising (5). There are 452 rehabilitation centers and 316 rehabilitation medicine specialists in the country (42). However, the distribution of rehabilitation centers and rehabilitation medicine specialists is also skewed toward the urban areas (43).

## Discussion

Since its discovery, AE is a rapidly evolving neurologic disease. The rapid advances of AE research in the past 10 years led to its ever-changing practice in terms of diagnosis and management (1). This precipitous growth necessitates commensurate endeavors among clinicians to keep up with the new knowledge. However, healthcare information inequality is also evident as lower and middle countries lack the capability to pursue research, the Philippines included. It is evident with the low number of studies and reports on the local experience on AE in the country that there is a need to pursue large-scale studies encompassing multicenter protocols focusing on the epidemiology, clinical course, morbidity and mortality rates, quality of life, and life expectancies of persons diagnosed with AE. The diagnosis of AE can be challenging since its clinical manifestations can be similar to other neuro-infectious and psychiatric disorders. Furthermore, objective evaluation requires the use of advanced facilities and diagnostic examinations to rule out other causes (1, 2). Due to the difficulty in establishing the diagnosis of AE and the lack of a robust registry system for patients with AE, the Philippines lacks epidemiologic data on AE in which underreporting, and misdiagnosis is a major concern. This in turn restricts the opportunity to increase awareness of AE and limits the establishment of national health care programs targeted to this condition.

Our review found that the health services offered in the country are insufficient to meet the needs of individuals with AE. Access to a neurologist has significant implications since AE is a complex and evolving disease. Furthermore, the recommended diagnostic tests are unavailable to most of the population limiting the opportunity for prompt recognition and treatment. Most of the treatment regimens for AE are expensive, only available in tertiary hospitals, and are purchased as an out-of-pocket expense. Access to neurorehabilitation, which is essential to manage long-term complications and improve functional capacity, is also limited by its cost and the limited number of rehabilitation centers and physiatrists alike. The topography of the Philippines further highlights the inequality of care between its citizens since most hospitals and practitioners capable of managing AE are concentrated in urbanized areas. The country also lacks access to ongoing clinical trials in AE as large-scale clinical trials for AE are limited due to its vast number of subtypes (5). Health care policies still do not acknowledge the presence of AE as an emerging neurologic disease as evidence by its absence in the medical case rate of PhilHealth. The allocated case rate of USD 432 under the category of other encephalitis is barely enough to cover the diagnostic evaluation for the disease let alone the therapeutic regimes and possible long-term care (13). This inadequate coverage of government-led health care financing led the majority of Filipinos to spend from their own pockets. These findings are in line with the recent studies addressing the gaps in the management of different neurologic diseases (Parkinson's disease, primary brain tumor, multiple sclerosis, epilepsy, stroke) in the Philippines (23, 33, 42–44).

**Figure 2:**
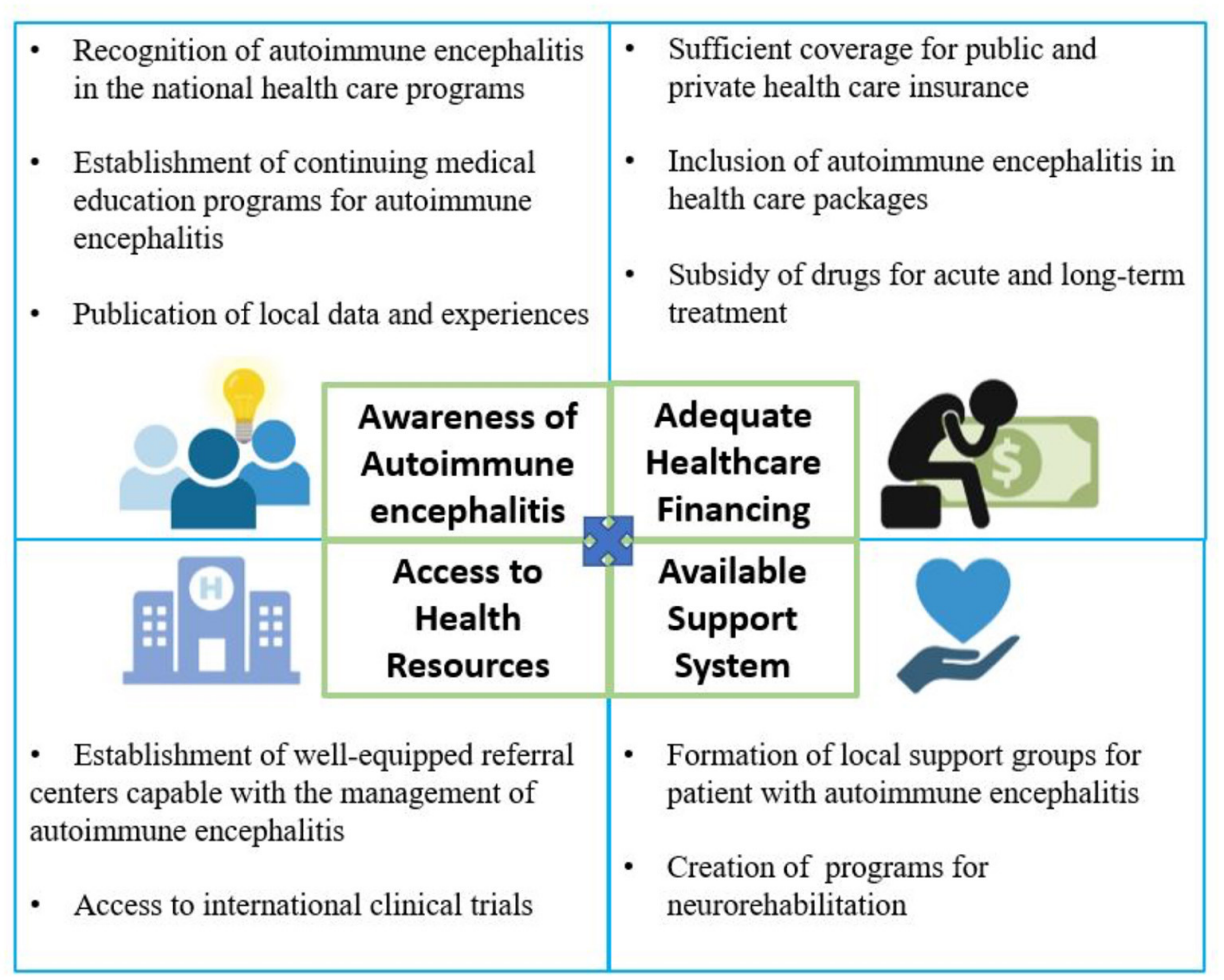
Conceptual framework of the gaps in the management of autoimmune encephalitis in the Philippines.

In general, the management of advanced neurologic diseases in the Philippines, AE included, presents with economic, governmental, and social challenges. Figure 2 summarizes these challenges together with the proposed recommendations to address these gaps. The awareness of AE is important for both societal and governmental programs to address these gaps. The next challenge is the access to comprehensive and timely diagnostic evaluation especially the access for the CSF and serum antibody testing. Problems with out-of-pocket expenditure can be addressed by promoting coverage from both public and private insurance institutions. Reducing the cost of cranial imaging is of utmost importance to facilitate access for initial evaluation. Institutional collaborations with accredited international laboratories coupled with adequate health financing present a viable option in addressing the lack of comprehensive CSF and serum antibody panel testing in the country. With the emerging evidence on the use of PLEX, IVIG, MPPT, rituximab, and cyclophosphamide, widespread availability of these drugs in government hospitals is recommended since they are already part of the national formulary. Lastly, establishing the practice of neuroimmunology in the Philippines can be promoted by supporting fellowship programs for neurologists outside the country.

## Limitations

This review may not have identified all relevant studies in the published, unpublished, and unreported literature despite our efforts to be comprehensive as possible. The keywords use and the search algorithm may have missed other relevant terms. Our review only included an article published in English and may have missed other studies published in a different language. Reported cases of AE throughout the Philippines are also subject to bias from underrecognition, misdiagnoses, underreporting, and/or publication. Furthermore, the pair of reviewers used their judgment to determine whether the studies have sufficiently met our criteria and are subject to reviewer bias.

To our knowledge, this is the first scoping review describing the gaps in the management of AE in the Philippines. Our study may have certain limitations in the access of articles especially on literature that is yet to be published and reports of local experiences of neurologists. The findings of our study promote awareness to patients, caregivers, clinicians, researchers, and legislators alike. The recognition of the treatment gaps in AE care in the Philippines strives to be the first step in promoting measures to improve health care delivery in the country.

## Data Availability Statement

The raw data supporting the conclusions of this article will be made available by the authors, without undue reservation.

## Author Contributions

All authors contributed to the study's conception and design. Material preparation, data collection, and analysis were performed by all of the authors. The first draft of the manuscript was written by GP and all authors commented on previous versions of the manuscript. All authors read and approved the final manuscript.

## Conflict of Interest

The authors declare that the research was conducted in the absence of any commercial or financial relationships that could be construed as a potential conflict of interest.

## Publisher's Note

All claims expressed in this article are solely those of the authors and do not necessarily represent those of their affiliated organizations, or those of the publisher, the editors and the reviewers. Any product that may be evaluated in this article, or claim that may be made by its manufacturer, is not guaranteed or endorsed by the publisher.

## Abbreviations

AE: Autoimmune Encephalitis
CSF: Cerebrospinal Fluid
DOH: Department of Health
EEG: Electroencephalogram
FDG-PET: Fluorodeoxyglucose Positron Emission Tomography
IVIG: Intravenous Immunoglobulin
MPPT: Methylprednisolone Pulse Therapy
MRI: Magnetic Resonance Imaging
PhilHealth: Philippine Health Insurance Corporation
PLEX: Plasma Exchange.
